# Psychometric evaluation and invariance of the Spanish version of the Block Fat Screener (BFS-E) in university students

**DOI:** 10.3389/fpsyg.2023.1055468

**Published:** 2023-03-27

**Authors:** Jacksaint Saintila, Wilter C. Morales-García, Yaquelin E. Calizaya-Milla, Percy G. Ruiz Mamani, Salomón Huancahuire-Vega, Sergio E. Calizaya-Milla, Cristian Ramos-Vera

**Affiliations:** ^1^Escuela de Medicina Humana, Universidad Señor de Sipán, Chiclayo, Peru; ^2^Escuela de Medicina Humana, Facultad de Ciencias de la Salud, Universidad Peruana Unión, Lima, Peru; ^3^Grupo de Investigación en Nutrición y Estilo de Vida, Universidad Peruana Unión, Lima, Peru; ^4^Escuela de Enfermería, Universidad Privada San Juan Bautista, Lima, Peru; ^5^Dirección General de Investigación, Universidad Peruana Unión, Lima, Peru; ^6^Research Area, Faculty of Health Sciences, Universidad César Vallejo, Lima, Peru

**Keywords:** fat consumption, psychometrics, factor analysis, questionnaire, university students

## Abstract

**Introduction:**

University students constantly face a number of health challenges related to an unhealthy diet, characterized by a high intake of saturated fats.

**Objective:**

This study aimed to analyze the psychometric properties of the Spanish version of the Block Fat Screener (BFS-E) food frequency questionnaire in a university population.

**Methods:**

An observational analytical study of instrumental type was carried out in 5608 Peruvian university students. Based on the Block Fat Screener questionnaire, a back-translation and cultural adaptation process was carried out. The validity of the questionnaire was determined through exploratory factor analysis (EFA) and confirmatory factor analysis (CFA), hypothesizing a unidimensional structure. For the determination of reliability, the alpha coefficients were considered; likewise, the ω and H coefficients were used to evaluate the construct. The model explained 63% of the cumulative variance.

**Results:**

The CFA confirmed the unidimensionality of the 16-item questionnaire with appropriate goodness-of-fit indicators; therefore, which model of the Peruvian version adequately fits the observed data. The values of the reliability coefficients were higher than 0.90, with ordinal α = 0.94, ω = 0.94, and H = 0.95.

**Conclusion:**

The Spanish version of the Block Fat Screener food frequency questionnaire presents adequate psychometric properties and is therefore a valid scale to quickly measure fat intake in university students in a Latin American context.

## Introduction

Non-communicable diseases (NCDs) represent one of the major public health problems in the world (Gowshall and Taylor-Robinson, 2018). They are responsible for 71% of the total number of deaths occurring each year; in fact, NCDs are the leading cause of death globally [World Health Organization (WHO), 2019]. Cardiovascular diseases (CVDs) are the leading cause of death among NCDs with 17.9 million deaths per year globally [World Health Organization (WHO), 2019]. Ischemic heart disease and stroke lead to the loss of more than 17 million lives (Organización Mundial de la Salud, 2012). In Peru, CVDs represent the main cause of death in the Peruvian population in general (EsSalud, 2019). A report estimated that 16% of the population over 20 years of age suffers from coronary heart disease and more than 2,000 die each year from some form of heart failure [World Hearth Federation/World Congress of Cardiology and Cardiology Health (WHF/WCC), 2016]. This reality seems to have repercussions in university students; a recent finding showed that the prevalence of cardiovascular risk factors in university students was 70.6% (Diaz-Salazar and Diaz-Salazar, 2021).

The presence of healthy dietary fats is an indicator of a healthy diet and is important for the proper functioning of cells, facilitating the utilization of fat-soluble vitamins, and promoting the bioavailability of carotenoids (Failla et al., 2014). However, excess saturated fats have been associated with the development of NCDs, particularly CVD (Astrup et al., 2020). Saturated fats raise serum cholesterol levels and can negatively impact cardiovascular health (Mozaffarian et al., 2009). Therefore, reducing saturated fat intake should be part of the essential measures to prevent NCDs. Reducing saturated fat consumption is already part of the policies of some non-governmental organizations, such as the American Heart Association, where it is recommended that apparently healthy adults limit fat intake to less than 7% of total daily calories (Budreviciute et al., 2020). Similarly, the World Health Organization in its Global Strategy on Diet and Physical Activity recommends changing the consumption of saturated fats to unsaturated fats and limiting their consumption in the diet (Waxman, 2004).

Becoming a university student can be exciting; however, many university students are constantly faced with trying to adapt to changes in academic workload, which have a negative impact on eating habits (Vidal et al., 2018). In fact, college students are a vulnerable group that constantly faces a number of health challenges related to an unhealthy diet, characterized by a high intake of saturated fats, sodium, and free sugars (Quiliche et al., 2021; Nuñez-Leyva et al., 2022). Several studies have reported unhealthy dietary practices among university students, including increased consumption of fast food and high-fat foods (Chourdakis et al., 2011; Mwafi et al., 2021; Saha et al., 2021). One study found that university students had significantly higher average intakes of total fat, saturated fat, and dietary cholesterol and lower intakes of polyunsaturated and monounsaturated fats (Chourdakis et al., 2011). The findings of a study that investigated the dietary intake of Swedish university students determined a significant increase in fat intake (Bergström et al., 2020). However, a study that evaluated dietary intake in Spanish university students found that the dietary habits of the respondents were characterized by a low intake of fats, fatty acids, and cholesterol (Correa-Rodríguez et al., 2018).

In general, male students consume high-fat foods more frequently (Saha et al., 2021). A study evaluating fat source consumption among university students found more frequent consumption of fatty products in the male population (Głodek and Gil, 2012). Possible factors influencing fatty food consumption among university students include factors such as taste, cost, accessibility, availability, environment, and location (Saha et al., 2021). Considering the role that saturated fats play in the body, they should be consumed in adequate and minimal amounts, since high consumption could have a negative impact not only on the health of students but also on the general population. Consequently, the evaluation of fat intake at the university stage should be part of NCD prevention strategies.

The Block Fat Screener food frequency questionnaire has been widely used as a quick and valid way to determine habitual dietary fat intake (Block et al., 1986, 2000; Caan et al., 1995; Di Noia et al., 2008; Martin-Biggers et al., 2015; Nutritionquest, 2022). However, reliability and validity testing was limited to judgments based on the final rating as high and low risk (Wenhold et al., 2014). In Peru, although several food frequency questionnaires (FFQ) have been developed and validated, to date there is no validated FFQ that can specifically estimate habitual fat intake in the university population. Taking into account that need, this study aims to translate into Spanish and adapt the Block Fat Screener food frequency questionnaire to the national university reality, evaluating the factorial structure (EFA and CFA) and reliability by means of the α ordinal, ω and H coefficients.

## Materials and methods

### Design and participants

This is an instrumental study, aimed at studying the psychometric properties of a scale (Ato et al., 2013). The number of participants was determined using Soper software (Soper, 2021) for structural equation models (SEM), we considered the number of observed (sixteen items) and a latent variable in the model, the size of the small effect (*λ* = 0.10), the desired probability (*α* = 0.05) and the statistical power level (1 − *β* = 0.80) considering a recommended minimum sample of 200.

### Instrument and data collection

An online survey was used to collect data between March and May 2021. The survey was available on a digital platform (Virtual classroom) implemented by Universidad Peruana Unión. Students found out about the research when they logged on to the virtual classroom home page of the university. Data were collected online due to social isolation measures implemented in response to the COVID-19 pandemic. The survey consisted of questions related to sociodemographic data such as age, sex, and dietary fat intake.

### Ethical considerations

Moreover, the survey contained information on informed consent, the purpose of the study, the confidential use of the data, the voluntariness of participation, and the duration. In addition, informed consent was obtained from the participants. Only when participants clicked on the *“Yes, I wish to participate”* option did they enter the survey, otherwise the web page window was automatically closed. The project was approved by the Research Ethics Committee of the Universidad Peruana Unión and registered under number 2021-CEUPeU-0009. It was also performed in accordance with the ethical criteria established in the Helsinki Declaration of 1975 and its subsequent modifications (Asocición Médica Mundial, 2013).

### Questionnaire

*Block Dietary Fat Screener:* It is a FFQ created by Block et al. (2000) and Nutritionquest (2022). It is made up of 17 items that include the main sources of fats. The questionnaire includes questions on frequently consumed high-fat foods (41 foods) and is designed to rank individuals with respect to their total habitual fat intake, using 5 response options (0 = 1 time per month or less, 1 = 2 to 3 times per month, 2 = 1 to 2 times per week, 3 = 3 to 4 times per week, and 4 = 5 to more times per week). A lower score corresponds to a very low fat intake and a higher score indicates a very high fat intake.

### Translation and cultural adaptation process

1. This questionnaire of habitual fat intake was translated into Spanish by two registered dietitians, experts in public health and fluent in both English and Spanish. An independent direct translation of the questionnaire into Spanish (Peru) was performed, following the guidelines established for translation and cross-cultural validation of psychometric instruments (Tsang et al., 2017).
2. The initial Spanish version was translated back into English by two independent Native American translators who were not familiar with the questionnaire, and the authors compared the two versions to determine cultural equivalence.
3. The Spanish version was evaluated by dietetic professionals. It was decided to merge items 8 “Margarine, butter or oil in the kitchen” and 9 “Eggs” from the original version and from this, the Spanish version of the 16-item BFS-E was developed. These two elements were merged due to the volume of consumption. For example, these foods are consumed in smaller proportions in all cases and are used in small quantities, with reduced volumes.
4. Finally, the instrument was applied to 9 university students as a focus group to evaluate its comprehension and readability. No modifications to the questionnaire were suggested and the final version was considered (Appendix A).

### Statistical analysis

Descriptive statistics were performed by calculating the mean, standard deviation, skewness, kurtosis, and corrected inter-test correlation analysis. For skewness and kurtosis, values between ±1.5 were considered adequate (Pérez and Medrano, 2010), and a corrected item-test correlation analysis was considered for item removal in case of r(i-tc) = <0.2 or multicollinearity (i-tc) = <0.2 (Pérez and Medrano, 2010).

To examine the factor structure of the BFS-E, an exploratory factor analysis (EFA) was performed by unweighted least squares, applying an oblique rotation (promax). To determine the optimal number of factors, parallel analysis was performed. The Kaiser-Meyer Olkin sample adequacy (KMO) and Bartlett’s test of sphericity were used to determine the adequacy of the EFA (Kaiser, 2016; Worthington and Whittaker, 2016). In turn, the confirmatory factor analysis (CFA) was performed using the “lavaan” package (Rosseel, 2012), hypothesizing the proposed unidimensional analysis. The WLSMV estimation method was used, as well as the goodness-of-fit indicators (Magaña et al., 2017). In this way, χ^2^ will be considered, in addition, the calculation of the magnitude of fit indices such as SRMR and RMSEA <0.05, also CFI and TLI > 0.90 were included (Kline, 2011; Raykov and Marcoulides, 2012). The convergent internal validity of the instrument was through the average variance extracted (AVE) considering >0.50 to argue that the items represent the construct (Fornell and Larcker, 1981a). As for the reliability estimation, the ordinal coefficient alpha (*α*), ω coefficient to assess the construct, and H coefficient (>0.70) were taken into account (Hunsley and Marsh, 2008; Ponterotto, 2009). Convergent validity was considered through calculation of the AVE, which accepts values greater than 0.50, since it indicates that more than 50% of the variance is due to its indicators (Fornell and Larcker, 1981b) and the permanence of the items was determined by taking into account factor loadings greater than 0.50 (*λ* > 0.50) (Hair et al., 2019). For discriminant validity it is expected that the AVE is greater than φ2 indicating that each individual factor is better explaining group membership than the difference between groups (AVE > φ^2^) (Fornell and Larcker, 1981b). Finally, factorial invariance was analyzed according to sex using a restrictive progressive step (Vandenberg and Lance, 2016). establishing the minimum differences between the reference model and the other models, taking into account the goodness of fit considering the CFI(ΔCFI) value ≤0.01 (Cheung and Rensvold, 2009). First, a reference or configural model (M1) was established, which allows the estimation of factor loadings, intercepts, and residuals. Second, metric invariance (M2), which consists of sequentially adding equality constraints between groups, was evaluated. Third, structural invariance was analyzed in which equality restrictions on the interfactor variances and covariances are added. The statistical analysis was carried out using the free software R 4.1.1 (R Foundation for Statistical Computing, Vienna, Austria).^1^

## Results

A total of 5,608 (46.7% men and 53.3% women) aged 18–36 years (*M* = 21.21 years and SD = 3.41) participated. The majority (70.2%) lived in urban areas. Regarding marital status, 94.7% were single and 5.3% were married. In addition, 32% were studying a health sciences degree, 30.78% were engineering and architecture faculty students, 27.2% business, and 10.1% were humanities faculty students. Finally, according to dietary regimen, 33.2% were omnivorous, 38.6% indicated that they were semi-vegetarian, 16.9% were lacto-ovo vegetarian, 7.2% were pesco vegetarian, and only 4.1% were vegan (see Table 1).

**Table 1:** Sociodemographic characteristics of telehealth program participants (*n* = 5,608).

Variable	*n* (%)
Age (*M* ± SD)	21.21 ± 3.41
*Gender*	
Male	2,619 (46.7)
Female	2,989 (53.3)
Place of residence	
Urban	3,938 (70.2)
Rural	1,671 (29.8)
Marital status	
Married	297 (5.3)
Single	5,311 (94.7)
Faculty of study	
Health sciences	1795 (32.0)
Engineering and architecture	1722 (30.7)
Business	1,526 (27.2)
Humanities	565 (10.1)
Dietary regimen	
Omnivorous	1861 (33.2)
Semi-vegetarian	2,163 (38.6)
Pesco-vegetarian	406 (7.2)
Lacto-ovo-vegetarian	949 (16.9)
Vegan	229 (4.1)

M, Mean; SD, standard deviation.

### Descriptive statistics of the items of the spanish version of the Block Fat Screener (BFS-E) questionnaire

Table 2 shows the descriptive statistics, where item 10 presented the lowest mean (*M* = 0.50) and item 3 presented the highest mean (*M* = 1.20). It was also observed that item 6 (Fried chicken) presented the highest variability (SD = 1.38), while the lowest item was item 16 (Pizza) (SD = 0.74). The skewness (g1) and kurtosis (g2) values fluctuating between ±1.5 indicated a multivariate non-normal distribution, therefore it was decided to use WLSMV (Pérez and Medrano, 2010). All corrected item-to-total correlations were greater than the acceptable limit of 0.20 (Pérez and Medrano, 2010).

**Table 2:** Descriptive statistics and reliability of the Spanish version of the BFS-E questionnaire.

Item	*M*	SD	Skew	Kurtosis	r-itc
1	0.85	1.16	1.33	0.88	0.61
2	0.85	1.1	1.17	0.50	0.67
3	1.20	1.08	0.62	−0.32	0.56
4	0.61	1.0	1.66	2.05	0.77
5	0.58	0.95	1.65	2.03	0.82
6	0.76	1.38	1.54	0.8	0.84
7	0.75	1.04	1.32	0.95	0.69
8	0.73	1.0	1.31	1.02	0.77
9	0.78	1.04	1.22	0.68	0.69
10	0.50	0.87	1.91	3.35	0.77
11	0.94	1.08	0.97	0.08	0.6
12	0.88	1.08	1.05	0.21	0.6
13	1.02	1.02	0.82	0.04	0.68
14	0.82	0.99	1.11	0.6	0.73
15	1.13	0.79	1.22	2.51	0.67
16	1.12	0.74	1.37	3.38	0.63

*M*, Mean; SD, standard deviation; r-itc, Correlation of item-total-corrected.

### Exploratory factor analysis

Parallel analysis (PA) indicated that a two factors model best represented the observed data, and the model explained 43% of the cumulative variance for the first factor and 21% for the second factor (Table 3). A unifactorial model found in the previous study was also tested (26.31). To determine whether the sample was adequate to apply the EFA, a Kaiser-Meyer-Olkin (KMO) sample adequacy measure was used, which showed a value of 0.95 and Barlett’s test of sphericity (*χ*2 = 34235.308, df = 120, *p* < 0.001) supported the unifactorial scale. Likewise, the factor loadings were greater than 0.50 and the AVE was adequate (AVE = 0.50).

**Table 3:** EFA of the unifactorial and two factors model of the BFS-E.

	Unifactorial			Two factors			
Item	F1	h2	u2	F1	F2	h2	u2
1	0.5	0.25	0.75	**0.43**	0.27	0.26	0.74
2	0.59	0.35	0.65	**0.48**	0.35	0.35	0.65
3	0.51	0.26	0.74	0.35	**0.38**	0.26	0.74
4	0.68	0.46	0.54	**0.68**	0.28	0.54	0.46
5	0.74	0.55	0.45	**0.75**	0.30	0.66	0.34
6	0.72	0.52	0.48	**0.73**	0.30	0.62	0.38
7	0.62	0.38	0.62	**0.47**	0.39	0.38	0.62
8	0.7	0.49	0.51	0.47	**0.51**	0.48	0.52
9	0.61	0.37	0.63	0.36	**0.51**	0.38	0.62
10	0.68	0.46	0.54	**0.53**	0.43	0.46	0.54
11	0.52	0.28	0.72	0.24	**0.51**	0.32	0.68
12	0.52	0.27	0.73	0.27	**0.48**	0.30	0.70
13	0.62	0.38	0.62	0.27	**0.63**	0.47	0.53
14	0.65	0.43	0.57	0.29	**0.66**	0.52	0.48
15	0.61	0.37	0.63	0.28	**0.60**	0.44	0.56
16	0.56	0.31	0.69	0.26	**0.54**	0.36	0.64

h2 = communality of each variable, u2 = specific variance of each variable. F1 = Factor 1; F2 = Factor 2.

### Evidence of validity and reliability

The CFA tested the two factors version proposed by the EFA, the statistical fit of the model was adequate, *χ*2 = 4,175, df = 103; CFI = 0.941, TLI = 0.931, RMSEA = 0.084 (IC 90%: 0,082-0,086), SRMR = 0.047. However, for convergent validity, the AVE value was 0.47 for F1 lower than accepted (AVE > 0.50) indicating that the items are not adequately measuring the construct or that there is redundancy in the items and 0.60 for F2. Furthermore, the square of the interfactor correlations (φ2) was lower than expected (AVE > φ2). Therefore, it was necessary to corroborate a unifactorial model, which indicated an adequate fit: *χ*2 = 3,163, df = 103; CFI = 0.956, TLI = 0.949, RMSEA = 0.073 (90% CI: 0.071–0.075), SRMR = 0.041. Likewise, the AVE value was 0.53, indicating that the factor is being measured consistently by the items (AVE > 0.50), therefore the unidimensional model fits the observed data adequately. In addition, the factor loadings (*λ*) of both models were greater than 0.50 (Table 4).

**Table 4:** Confirmatory Factor Analysis, one-factor model, two-factor model, and reliability.

	Unifactorial	Two factors	
Item	F1 (*λ*)	F1 (*λ*)	F12(*λ*)
P1	0.614	0.58	
P2	0.668	0.8	
P3	0.559	0.72	
P4	0.784	0.62	
P5	0.846	0.62	
P6	0.865	0.7	
P7	0.687	0.75	
P8	0.772	0.7	
P9	0.698	0.65	
P10	0.77		0.64
P11	0.601		0.69
P12	0.601		0.81
P13	0.681		0.87
P14	0.729		0.88
P15	0.681		0.71
P16	0.631		0.79
AVE	0.53	0.47	0.6
F1	–	–	**0.757**
F2	–	0.871	–
*α*_ordinal_	0.94	0.84	0.84
*ω*	0.94	0.84	0.84
*H*	0.95	0.84	0.84

F1 = Factor 1; F2 = Factor 2; Ordinalα: Ordinal alpha; λ = Factor loading; AVE: average variance extracted; below the diagonal: interfactor correlations; above the diagonal: variance shared between factors (AVE > φ2).

### Internal consistency analysis

As for internal consistency, it was evaluated by ordinal alpha, McDonald’s Omega, and H coefficient (Table 1), and the values of the reliability coefficients were acceptable (Hancock and Mueller, 2001; Gadermann et al., 2019), as shown, values higher than 0.90 were obtained, with ordinal *α* = 0.94, *ω* = 0.94 and *H* = 0.95 (Table 2).

## Discussion

The university student population faces health challenges related to an unhealthy diet (Quiliche et al., 2021; Nuñez-Leyva et al., 2022). In the case of Peruvian university students, more than 40% reported having a high fat intake (Vidal et al., 2018), which could put your health at risk. Therefore, there is a need to identify these students and implement strategies that promote the reduction of fat intake, which requires simple, inexpensive, fast, and valid tools that can provide a snapshot of the diet and fat intake of college students. This study aimed to analyze the psychometric properties of the BFS-E and to demonstrate the first evidence of validity and reliability, obtaining an adapted version that can be used to implement studies to increase its predictive capacity. In this sense, it is valuable to make linguistic adaptations in specific cultural contexts, as is the case of the Peruvian university population (Squires et al., 2013).

The BFS-E is in an experimental phase and the satisfactory evidence found in this psychometric evaluation feeds the implementation of further studies. Although there is no previous research evaluating a fat intake measurement instrument in the academic setting, previous studies consider the need for culturally adapted tools to comprehensively capture dietary intake and allow for greater accuracy in measurement (Hebert et al., 2008; Kandola et al., 2016). Therefore, it is necessary to replicate the study to strengthen the psychometric properties. In the descriptive analysis, it was verified that the homogeneity indices of all items were correlated above 0.20, which suggests that all items belong to the construct (Rex, 2015). The EFA was established given the lack of precedents in Latin America; therefore, it was considered appropriate to explore the structure.

Parallel analysis (PA) indicated the existence of two factors; however, we considered evaluating the unifactorial model proposed above (Hunsley and Marsh, 2008; Ponterotto, 2009). The CFA was used to evaluate the goodness-of-fit indices of the models. The results indicate that the unidimensional model has adequate goodness-of-fit indices. Likewise, in terms of convergent validity, the AVE indicates that 50% of the variance is due to the 16 items, and the factor loadings were greater than 0.50 (Worthington and Whittaker, 2006). Unlike the two factors model, which presented a high interfactorial correlation, that is, an overparameterization or specification of parameters that should not be there, since they would configure a general dimension (Fornell and Larcker, 1981a; Saris et al., 2009).

On the other hand, in terms of reliability, different reliability indices suitable for the models were calculated. The magnitude found is within the appropriate limits for instruments for research purposes (ordinal α, ω, and H > 0.70). The ordinal α exceeds the suggested 0.80 in unidimensional factor (Campo-Arias and Oviedo, 2008). Coefficients ω and H were also evaluated since these coefficients evaluate latent variables and are considered better estimators as opposed to alpha, which tends to underestimate reliability (Dunn et al., 2014). The ω was similar to the ordinal α, while the H values were slightly higher than ω. The BFS-E measurement is valid and reliable; therefore, it would be useful in providing stable results for pre-and post-test designs or empirical studies.

It is important to consider that the present study examined invariance in relation to sex. The factor structure at the four levels (configural, metric, strict, and scalar) remained stable for both males and females, therefore, the items measure similarly the latent trait for both groups (Campo-Arias and Oviedo, 2008; Dunn et al., 2014).

Therefore, having this quick-to-use instrument validated in Spanish and having items that measure both groups similarly will allow the use of this instrument for future comparison studies. Likewise, will provide immediate feedback to the university student on his or her fat intake status and give the physician or nutritionist the opportunity to make more in-depth evaluations based on this first filter. In addition, this questionnaire/scale is designed to obtain a valid measure of fat intake in much less time than that required by traditional food records or extensive food frequency questionnaires. The assessment can be completed and scored in less than 5 min and does not require complicated calculations. Finally, the scale includes the main sources of fat in the diet of the Peruvian population.

### Limitations

Some limitations are present in this research, because given the non-probabilistic nature of the sample, it does not allow generalizations to be made to other populations; however, considering the sample size, the results of the internal structure and reliability, it can be considered a robust instrument. It should also be noted that temporal stability analyses were not performed and other age groups with similar characteristics in Latin America were not explored, since the psychometric objective of the study was the initial evaluation of the BFS-E in Peru. In addition, it was not possible to analyze gender differences using invariance. Future research should diversify the current sample to evaluate differences in other cultural contexts. Moreover, the use of probabilistic sampling is suggested to provide the possibility of extrapolating the results to the total population.

## Conclusion

In conclusion, the BFS-E has shown adequate psychometric properties and gender invariance in the population of Peruvian university students, therefore it can be a rapid measurement instrument that assesses dietary fat intake. However, it is important to emphasize that this is an initial study of BFS-E in a Latin American population with conclusive results.

## Data availability statement

The raw data supporting the conclusions of this article will be made available by the authors, without undue reservation.

## Ethics statement

The studies involving human participants were reviewed and approved by the Research Ethics Committee of the Universidad Peruana Unión. The patients/participants provided their written informed consent to participate in this study.

## Author contributions

JS, WM-G, and YC-M participated in the conceptualization of the idea. PGRM, SH-V, and CR-V were in charge of the methodology and software. WM-G, YC-M, PGRM, and CR-V were responsible for validation, formal analysis, and research. WM-G and SC-M commissioned the data curation and resources. JS, WM-G, SH-V, and SC-M carried out the writing of the first draft, review and editing, visualization, and supervision. All authors have read and approved the final version of the manuscript.

## Funding

This work was supported in part by the Universidad Señor de Sipán of Chiclayo, Perú (RESOLUCIÓN DE DIRECTORIO N° 015-2022/PD-USS) and Universidad Peruana Unión, Perú (Resolución N° 2556-2021/UPeU-CU).

## Conflict of interest

The authors declare that the research was conducted in the absence of any commercial or financial relationships that could be construed as a potential conflict of interest.

## Publisher’s note

All claims expressed in this article are solely those of the authors and do not necessarily represent those of their affiliated organizations, or those of the publisher, the editors and the reviewers. Any product that may be evaluated in this article, or claim that may be made by its manufacturer, is not guaranteed or endorsed by the publisher.

## Footnotes

^1^http://www.R-project.org

## Appendix A

Block Fat Screener (BFS-E) – Spanish version

Cribado de grasas de Block

0 = 1 vez al mes o menos

1 = de 2 a 3 veces al mes

2 = 1 a 2 veces a la semana

3 = 3 a 4 veces a la semana

4 = 5 a más veces a la semana

**Table:**

¿Aproximadamente con qué frecuencia consume los siguientes alimentos?					
Ítems	0	1	2	3	4
1. Hamburguesas, carne molida, o tacos					
2. Carne de res o cerdo, como filetes, asados, costillas o en bocadillos					
3. Pollo frito					
4. Hot dogs o salchicha					
5. Embutidos, fiambres, o jamón (no bajo en grasas)					
6. Tocino o salchicha en el desayuno					
7. Aderezos para ensaladas (no bajos en grasa)					
8. Margarina, mantequilla o mayonesa para cocinar y utilizar sobre pan o papas					
9. Huevos (solo claras de huevo)					
10. Pizza					
11. Queso, o queso para untar (no bajo en grasa)					
12. Leche entera					
13. Papas fritas					
14. Chips de maíz, papas fritas paquete, palomitas de maíz, o galletas saladas?					
15. Donas, pasteles, o galletas (no bajas en grasa)?					
16. Helado que no tenga grasa (helados de hierro, de pula de frutas, entre otros).					

**Table:**

Escalas de Valoración		
Características	Nivel	Intervalo
General	Bajo	0–6
	Medio	7–17
	Alto	18–64
Femenino	Bajo	0–6
	Medio	7–15
	Alto	16–64
Masculino	Bajo	0–7
	Medio	8–19
	Alto	20–64

## References

[ref1] Asocición Médica Mundial. (2013). Declaración de Helsinki de la AMM–Principios éticos para las investigaciones médicas en seres humanos. Ferney-Voltaire WMA–The World Medical Association.

[ref2] Astrup A. Magkos F. Bier D. M. Brenna J. T. de Oliveira Otto M. C. Hill J. O.. (2020). Saturated fats and health: a reassessment and proposal for food-based recommendations. J. Am. Coll. Cardiol. 76, 844–857. doi: 10.1016/j.jacc.2020.05.077, PMID: 32562735

[ref3] Ato M. López J. J. Benavente A. (2013). Un sistema de clasificación de los diseños de investigación en psicología. An. Psicol. 29, 1038–1059. doi: 10.6018/analesps.29.3.178511

[ref4] Bergström M. Håkansson A. Blücher A. Andersson H. S. (2020). From carbohydrates to fat: trends in food intake among Swedish nutrition students from 2002 to 2017. PLoS One 15:e0228200. doi: 10.1371/journal.pone.0228200 31990946 PMC6986719

[ref5] Block G. Gillespie C. Rosenbaum E. H. Jenson C. (2000). A rapid food screener to assess fat and fruit and vegetable intake. Am. J. Prev. Med. 18, 284–288. doi: 10.1016/S0749-3797(00)00119-7, PMID: 10788730

[ref6] Block G. Hartman A. M. Dresser C. M. Carroll M. D. Gannon J. Gardner L. (1986). A data-based approach to diet questionnaire design and testing. Am. J. Epidemiol. 124, 453–469. 3740045 10.1093/oxfordjournals.aje.a114416

[ref7] Budreviciute A. Damiati S. Sabir D. K. Onder K. Schuller-Goetzburg P. Plakys G.. (2020). Management and prevention strategies for non-communicable diseases (NCDs) and their risk factors. Front. Public Heal. 8:574111. doi: 10.3389/fpubh.2020.574111, PMID: 33324597 PMC7726193

[ref8] Caan B. Coates A. Schaffer D. (1995). Variations in sensitivity, specificity, and predictive value of a dietary fat screener modified from Block et al. J. Am. Diet. Assoc. 95, 564–568. 7722191 10.1016/S0002-8223(95)00153-0

[ref9] Campo-Arias A. Oviedo H. C. (2008). Propiedades psicométricas de una escala: La consistencia interna. Rev. Salud Publica. 10, 831–839. doi: 10.1590/S0124-00642008000500015, PMID: 19360231

[ref10] Cheung G. W. Rensvold R. B. (2009). Evaluating goodness-of-fit indexes for testing measurement invariance. Struct. Equ. Model. A Multidiscip. J. 9, 233–255. doi: 10.1207/S15328007SEM0902_5

[ref11] Chourdakis M. Tzellos T. Pourzitaki C. Toulis K. A. Papazisis G. Kouvelas D. (2011). Evaluation of dietary habits and assessment of cardiovascular disease risk factors among Greek university students. Appetite 57, 377–383. doi: 10.1016/j.appet.2011.05.314, PMID: 21651931

[ref12] Correa-Rodríguez M. Pocovi G. Schmidt-RioValle J. González-Jiménez E. Rueda-Medina B. (2018). Assessment of dietary intake in Spanish university students of health sciences. Endocrinol. Diabetes Nutr. 65, 265–273. doi: 10.1016/j.endinu.2018.01.005 29599102

[ref13] Di Noia J. Schinke S. P. Contento I. R. (2008). Dietary fat intake among urban, African American adolescents. Eat. Behav. 9, 251–256. doi: 10.1016/j.eatbeh.2007.07.006, PMID: 18329605 PMC2291026

[ref14] Diaz-Salazar W. Diaz-Salazar W. (2021). Cardiovascular risk factors in students of the Universidad particular de Chiclayo. Rev. Cuba. Tecnol. Salud. 12, 155–162.

[ref15] Dunn T. J. Baguley T. Brunsden V. (2014). From alpha to omega: a practical solution to the pervasive problem of internal consistency estimation. Br. J. Psychol. 105, 399–412. doi: 10.1111/bjop.12046, PMID: 24844115

[ref16] EsSalud (2019). Enfermedades al corazón son primera causa de muerte en adultos. EsSalud Lima, Perú

[ref17] Failla M. L. Chitchumronchokchai C. Ferruzzi M. G. Goltz S. R. Campbell W. W. (2014). Unsaturated fatty acids promote bioaccessibility and basolateral secretion of carotenoids and α-tocopherol by Caco-2 cells. Food Funct. 5, 1101–1112. doi: 10.1039/C3FO60599J, PMID: 24710065

[ref18] Fornell C. Larcker D. F. (1981a). Evaluating structural equation models with unobservable variables and measurement error. J. Mark. Res. 18, 39–50. doi: 10.1177/002224378101800104

[ref19] Fornell C. Larcker D. F. (1981b). Structural equation models with unobservable variables and measurement error: algebra and statistics. J. Mark. Res. 18, 382–388. doi: 10.1177/002224378101800313

[ref20] Gadermann A. M. Guhn M. Zumbo B. D. (2019). Estimating ordinal reliability for likert-type and ordinal item response data: a conceptual, empirical, and practical guide. Pract. Assessment Res. Eval. 17:3. doi: 10.7275/n560-j767

[ref21] Głodek E. Gil M. (2012). Evaluation of the nutrition model in students of university in Rzeszow. Rocz. Państw. Zakł. Hig. 63, 313–317. 23173336

[ref22] Gowshall M. Taylor-Robinson S. (2018). The increasing prevalence of non-communicable diseases in low-middle income countries: the view from Malawi. Int. J. Gen. Med. 11, 255–264. doi: 10.2147/IJGM.S157987, PMID: 29988742 PMC6029598

[ref23] Hair JF Black WC Babin BJ Anderson RE. (2019) Multivariate Data Analysis. 8th. North Way: Cengage Learning

[ref24] Hancock G. R. Mueller R. O. (2001). “Rethinking construct reliability within latent variable systems” in Structural Equation Modeling: Present and Future—A Festschrift in Honor of Karl Joreskog. eds. Cudeck R. Toit S. D. Soerbom D. (Lincolnwood, IL: Scientific Software International), 195–216.

[ref25] Hebert J. R. Hurley T. G. Peterson K. E. Resnicow K. Thompson F. E. Yaroch A. L.. (2008). Social desirability trait influences on self-reported dietary measures among diverse participants in a multicenter multiple risk factor trial. J. Nutr. 138, 226S–S234. doi: 10.1093/jn/138.1.226S 18156429

[ref26] Hunsley J Marsh EJ. (2008). Developing criteria for evidence-based assessment: an introduction to assessment that work. In: Hunsley J Marsh EJ, editors. A Guide to Assessments that Work. Hunsley J. Marsh E.J., Oxford University Press: Oxford, UK p. 3–14.

[ref27] Kaiser H. F. (2016). The application of electronic computers to factor analysis. Educ. Psychol. Meas. 20, 141–151. doi: 10.1177/001316446002000116

[ref28] Kandola K. Sandhu S. Tang T. (2016). Immigration and dietary patterns in south Asian Canadians at risk for diabetes. J. Diabetes Complicat. 30, 1462–1466. doi: 10.1016/j.jdiacomp.2016.08.003, PMID: 27591030

[ref29] Kline RB (2011) Principles and Practice of Structural Equation Modeling. New York: Guilford Press

[ref30] Magaña D. E. M. Aguilar N. M. Vázquez J. M. R. (2017). Análisis Factorial Confirmatorio para medir las limitantes percibidas en el pregrado para el desarrollo de actividades de investigación. Nov. Sci. 9, 515–536. doi: 10.21640/ns.v9i18.838

[ref31] Martin-Biggers J. Koenings M. Quick V. Abbot J. M. Byrd-Bredbenner C. (2015). Appraising nutrient availability of household food supplies using Block dietary screeners for individuals. Eur. J. Clin. Nutr. 69, 1028–1034. doi: 10.1038/ejcn.2015.30 25804271

[ref32] Mozaffarian D. Aro A. Willett W. C. (2009). Health effects of trans-fatty acids: experimental and observational evidence. Eur. J. Clin. Nutr. 63, S5–S21. doi: 10.1038/sj.ejcn.1602973, PMID: 19424218

[ref33] Mwafi N. R. Al-Rawashdeh I. M. Al-Kubaisy W. A. Q. Ezzat W. R. Al-Qazaqi R. A. Salameh M. H. (2021). Prevalence and factors related to obesity and fast food consumption among Mutah University students, Jordan. J. Pak. Med. Assoc. 71, 1608–1612. doi: 10.47391/JPMA.274, PMID: 34111082

[ref34] Nuñez-Leyva R. E. Lozano-López T. E. Calizaya-Milla Y. E. Calizaya-Milla S. E. Saintila J. (2022). Excess weight and body fat percentage associated with waist circumference as a cardiometabolic risk factor in university students. Scientifica 1310030. doi: 10.1155/2022/1310030 35036024 PMC8754593

[ref35] Nutritionquest (2022). Block Dietary Data Systems Fat Screener [Internet].Available at: https://www.nutritionquest.com/wellness/free-assessment-tools-for-individuals/fruit-vegetable-fiber-screener/

[ref36] Organización Mundial de la Salud (2012). Día mundial del corazón: Enfermedades Cardiovasculares causan 1,9 millones de muertes al año en las Américas. Available at: https://www.paho.org/es/noticias/29-9-2021-enfermedades-corazon-siguen-siendo-principal-causa-muerte-americas

[ref37] Pérez E. R. Medrano L. (2010). Análisis factorial exploratorio: bases conceptuales y metodológicas. Rev. Argent Cienc. Comport. 2, 58–66.

[ref38] Ponterotto J. G. (2009). Charter RA. Statistical extensions of ponterotto and ruckdeschel’s (2007) reliability matrix for estimating the adequacy of internal consistency coefficients. Percept. Mot. Skills 108, 878–886. doi: 10.2466/pms.108.3.878-886, PMID: 19725323

[ref39] Quiliche R. B. Turpo-Chaparro J. Torres J. H. Saintila J. Ruiz Mamani P. G. (2021). Overweight and obesity, body fat, waist circumference, and anemia in Peruvian university students: a cross-sectional study. Md Akim a, editor. J. Nutr. Metab. 2021, 1–9. doi: 10.1155/2021/5049037, PMID: 34925917 PMC8674050

[ref40] Raykov T. Marcoulides GA. (2012). A First Course in Structural Equation Modeling. New Jersey, London: Routledge

[ref41] Rex BK. (2015) Principles and Practices of Structural Equation Modelling. 4th. The Guilford Press New York, NY 1–554

[ref42] Rosseel Y. (2012). lavaan: an R package for structural equation modeling. J. Stat. Softw. 48, 1–36. Available at: https://www.jstatsoft.org/index.php/jss/article/view/v048i02

[ref43] Saha S. Al Mamun M. A. Kabir M. R. (2021). Factors affecting fast food consumption among college students in South Asia: a systematic review. J. Am. Coll. Nutr. 41, 626–636. doi: 10.1080/07315724.2021.1940354 34156900

[ref44] Saris W. E. Satorra A. van der Veld W. M. (2009). Testing structural equation models or detection of misspecifications? Struct. Equ. Model A Multidiscip. J. 16, 561–582. doi: 10.1080/10705510903203433

[ref45] Soper D. (2021). A-Priori Sample Size Calculator for Structural Equation Models [Software]. Available at: http://wwwdanielsopercom/statcalc

[ref46] Squires J. E. Hayduk L. Hutchinson A. M. Cranley L. A. Gierl M. Cummings G. G.. (2013). A protocol for advanced psychometric assessment of surveys. Nurs. Res. Pract. 2013, 1–8. doi: 10.1155/2013/156782, PMID: 23401759 PMC3562582

[ref47] Tsang S. Royse C. F. Terkawi A. S. (2017). Guidelines for developing, translating, and validating a questionnaire in perioperative and pain medicine. Saudi J Anaesth 11, S80–S89. doi: 10.4103/sja.SJA_203_17, PMID: 28616007 PMC5463570

[ref48] Vandenberg R. J. Lance C. E. (2016). A review and synthesis of the measurement invariance literature: suggestions, practices, and recommendations for organizational research 3, 4–70. doi: 10.1177/109442810031002,

[ref49] Vidal E. J. Alvarez D. Martinez-Velarde D. Vidal-Damas L. Yuncar-Rojas K. A. Julca-Malca A.. (2018). Perceived stress and high fat intake: a study in a sample of undergraduate students. PLoS One 13:e0192827. doi: 10.1371/journal.pone.0192827 29522535 PMC5844534

[ref50] Waxman A. (2004). Who global strategy on diet, physical activity and health. Food Nutr. Bull. 25, 292–302. doi: 10.1177/156482650402500310 15460274

[ref51] Wenhold F. A. M. MacIntyre U. E. Rheeder P. (2014). Reliability and validity of a modified MEDFICTS dietary fat screener in South African schoolchildren are determined by use and outcome measures. J. Acad. Nutr. Diet. 114, 870–880. doi: 10.1016/j.jand.2014.01.005, PMID: 24613420

[ref52] World Hearth Federation/World Congress of Cardiology and Cardiology Health (WHF/WCC) (2016). El costo de las enfermedades cardiacas en América Latina. WHF/WCC México City

[ref53] World Health Organization (WHO). (2019). Noncommunicable Diseases: Mortality. Geneva: WHO.

[ref54] Worthington R. L. Whittaker T. A. (2006). Scale development research. Couns. Psychol. 34, 806–838. doi: 10.1177/0011000006288127

[ref55] Worthington R. L. Whittaker T. A. (2016). Scale development research: a content analysis and recommendations for best practices. Couns. Psychol. 34, 806–838. doi: 10.1177/0011000006288127

